# Microfluidics for Synthesis of Peptide-Based PET Tracers

**DOI:** 10.1155/2013/839683

**Published:** 2013-10-31

**Authors:** Yang Liu, Mei Tian, Hong Zhang

**Affiliations:** ^1^Department of Nuclear Medicine, Second Affiliated Hospital of Zhejiang University School of Medicine, 88 Jiefang Road, Hangzhou, Zhejiang 310009, China; ^2^Zhejiang University Medical PET Center, Zhejiang University, 88 Jiefang Road, Hangzhou, Zhejiang 310009, China; ^3^Institute of Nuclear Medicine and Molecular Imaging, Zhejiang University, 88 Jiefang Road, Hangzhou, Zhejiang 310009, China; ^4^Key Laboratory of Medical Molecular Imaging of Zhejiang Province, 88 Jiefang Road, Hangzhou, Zhejiang 310009, China

## Abstract

Positron emission tomography (PET) is a powerful noninvasive tool for acquisition of the physiological parameters in human and animals with the help of PET tracers. Among all the PET tracers, radiolabeled peptides have been widely explored for cancer-related receptor imaging due to their high affinity and specificity to receptors. But radiochemistry procedures for production of peptide-based PET tracers are usually complex, which makes large-scale clinical studies relatively challenging. New radiolabeling technologies which could simplify synthesis and purification procedures, are extremely needed. Over the last decade, microfluidics and lab-on-a-chip (LOC) technology have boomed as powerful tools in the field of organic chemistry, which potentially provide significant help to the PET chemistry. In this minireview, microfluidic radiolabeling technology is described and its application for synthesis of peptide-based PET tracers is summarized and discussed.

## 1. Introduction

Positron emission tomography (PET) is increasingly being used for *in vivo *biochemical, physiological, and pharmacological process visualization and is also routinely used for screening, diagnosing, and staging of cancer. With the help of PET tracers and PET scanners, physiological parameters (like blood flow, metabolism, receptor properties, drug distribution, and gene expression) in the living human and animal bodies could be studied noninvasively. These PET tracers, including small molecules, peptides, antibodies, are radiolabeled with short-lived radioisotopes. Among these tracers, radiolabeled peptides have been widely explored for cancer imaging due to their high affinity and specificity to many kinds of cancer-related receptors. Peptide-based PET tracers have the following advantages: (1) peptides usually have very high affinity and specificity to the target (receptor); (2) compared to biomacromolecules like antibodies, peptides are easy to synthesize and characterize; (3) rapid clearance from the blood and nontarget tissues. A lot of peptide-based PET tracers are undergoing clinical trials, like the ^18^F-labeled RGD and ^68^Ga-labeled Octreotide [1, 2]. However, large-scale clinical studies and applications are relatively challenging due to the complex radiochemistry procedures for peptide-based PET tracers, especially for ^18^F-labeled tracers which usually require a laborious and time-consuming multistep process. Radiochemists are continually working on the development of new methods and technologies for the preparation of PET tracers. 

During the last several decades, microfluidics and lab-on-a-chip (LOC) technologies have boomed as powerful tools in the field of organic chemistry, showing characteristics like enhanced heat and mass transfer, reduction of reagent consuming and hazardous waste, which potentially provide significant help to the PET chemistry. Applications of microfluidic chemistry in radiopharmaceutical synthesis have also drawn increasing attention [5–9]. Microfluidic system could increase the overall efficiency of radiolabeling reaction remarkably. ^18^F-FDG production by the microfluidic system has been successfully demonstrated [10], and a lot of other PET tracers have been produced successfully. Compared with small molecules, synthesis of peptide-based PET tracers requires milder reaction conditions and strict chromatographic purification. Microfluidic system allows lower precursor consumption which simplified the purification, and the enhanced heat and mass transfer in microfluidic reactors can provide higher labeling yields under milder reaction conditions, so microfluidic system has good potential to play an important role in production of peptide-based PET tracers. 

In this minireview, microfluidic radiolabeling technology is described and its application for synthesis of peptide-based PET tracers is summarized and discussed. 

## 2. Peptide-Based PET Tracers

Biological active peptides are involved in many biochemical processes, like immune response and information transmission, and they play important roles in cellular communication and cell proliferation. On the other hand, a variety of receptors are found to be expressed on the membrane and possess very high affinity to specific peptides. Particular receptors are often massively overexpressed in cancer tissues, so peptide ligands could be utilized as targeting tools for PET imaging. Representative targets for peptide-based PET tracers are listed in Table 1.

Common radioisotopes used for peptide radiolabeling are listed in Table 2; half-lives of these radioisotopes (^18^F: *t*_1/2_ = 109.8 min, ^68^Ga: *t*_1/2_ = 67.6 min, ^64^Cu: *t*_1/2_ = 12.8 h) are suitable for the pharmacokinetics of most peptides. ^18^F is one of the most widely used radionuclides for diagnostic PET imaging because of its unique nuclear and chemical properties [15]. ^18^F-labeling of peptides can be achieved via prosthetic groups, such as N-succinimidyl-4-^18^F-fluorobenzoate (^18^F-SFB) [16] and 4-nitrophenyl 2-^18^F-fluoropropionate (^18^F-NFP) [17]. In recent years, a facile, 1-step Al^18^F method has been developed and demonstrated as a very promising method for radiofluorination of peptides, which does not require on-site cyclotron by use of the commonly available sodium ^18^F-fluoride solutions [18–20]. ^68^Ga is available from an in-house generator rendering ^68^Ga radiopharmacy independent of an on-site cyclotron, which is a big advantage for clinical use. ^68^Ga-labeled peptides have been developed for the targeting of somatostatin receptors, the melanocortin 1 receptor, the bombesin receptor, HER2 receptor and so forth [21, 22]. ^64^Cu is another widely used metallic positron emitter, which can be produced on a large scale with a medical cyclotron, and the half-life (12.7 h) and decay properties make it an ideal radioisotope for PET imaging and radiotherapy [23, 24]. Besides, coordination chemistry of copper is well established and a wide variety of chelator systems are available. ^64^Cu-labeled peptides have also been developed for the targeting of a variety of receptors.

**Figure 1:**
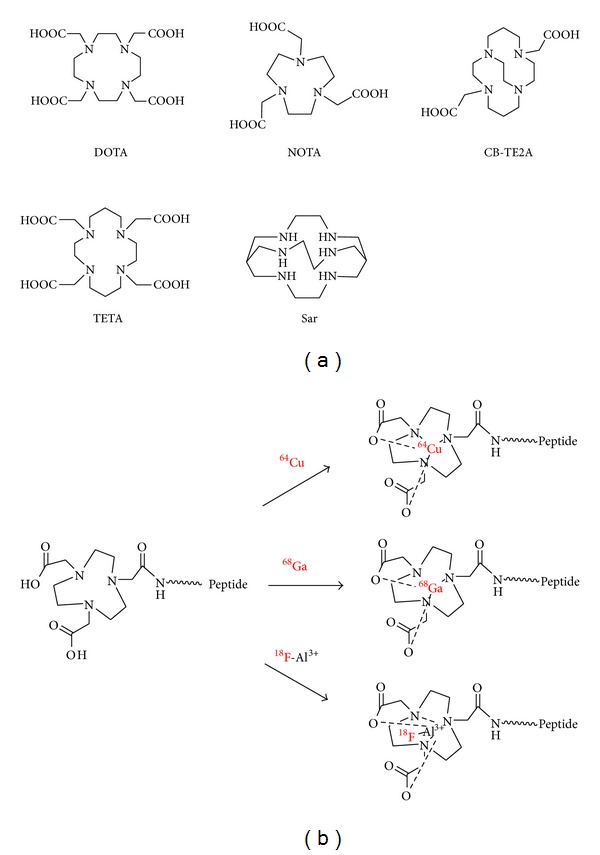
Basic bifunctional chelators (a) and schematic procedure for radiolabeling of peptides (b).

**Figure 2:**
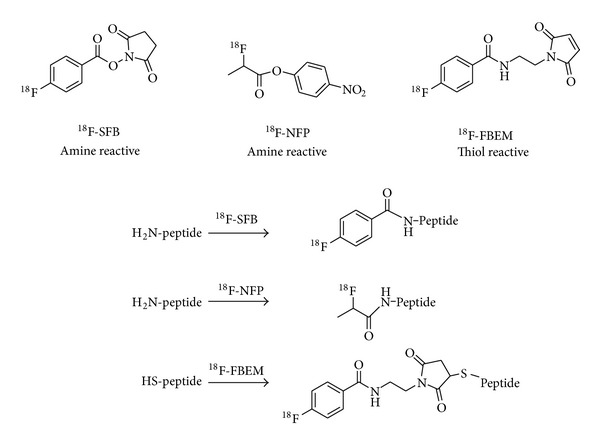
Representative prosthetic groups for radiolabeling of peptides.

Conventional methods for radiolabeling of peptides can be divided into two catalogs: labeling with radiometals via chelation chemistry [25] (Figure 1) and labeling through prosthetic groups [26] (Figure 2). For example, the BBN peptide and its analogues have been radiolabeled with various radionuclides including ^64^Cu, ^68^Ga, and ^18^F for GRPR-related cancer imaging [27–30]. In addition to the above two methods, direct radiolabelling of peptide analogues with a leaving group with ^18^F-fluoride was also reported [31]. Generally speaking, labeling through prosthetic groups is more time consuming, and labeling with radiometals via chelation chemistry is straightforward and usually has higher labeling yields. But some chelation reactions also require high temperature and long reaction time in order to get high yields. New technologies are always desired to improve the radiolabeling efficacy and efficiency.

## 3. Microfluidic Reactors

Microfluidic reactors, which generally consist of a network of micron-sized channels (typically 10–500 *μ*m) embedded in a glass, metal or plastic solid substrate, have already found broad applications in the fields of organic synthesis [32] and biomolecular labeling [33]. The basic aspects of microfluidic reactors have been well summarized in other reviews [34–37]. In recent years, multistep synthesis has been performed on multistep continuous-flow synthesis systems [38–40], which could be utilized as promising tools for the multistep synthesis and purification of radiopharmaceuticals for PET.

**Figure 3:**
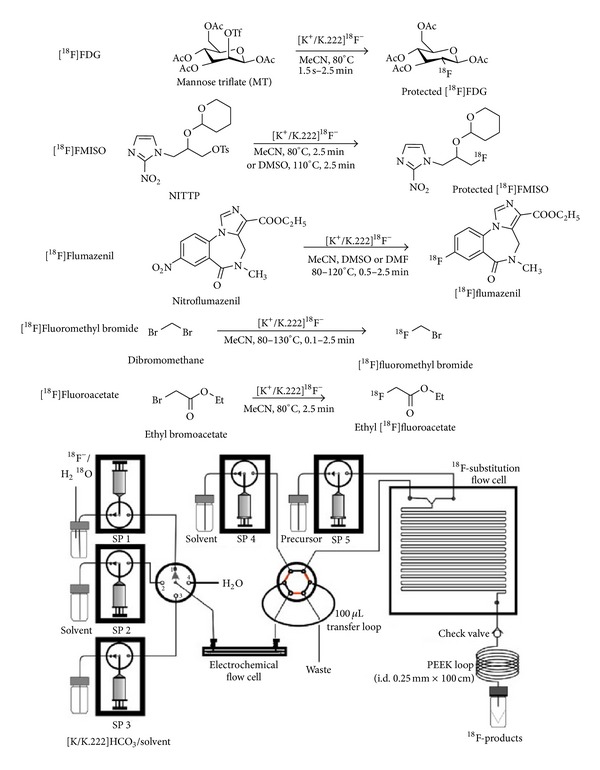
Microfluidic platform showing reaction setup using an electrochemical concentration chip and a reaction flow cell. ^18^F-fluorination yields for the four ^18^F-labeled compounds (protected ^18^F-FDG: 98%, protected ^18^F-FMISO: 80%, ^18^F-flumazenil: 20%, ^18^F-fluoromethyl bromide: 60%) were comparable to or higher than those obtained by conventional means (reproduced from [3] with permission from Elsevier).

Figure 3 showed representative microfluidic devices and the PET tracers synthesized [3]. Low precursor and reagent consumption, efficient heat transfer, and enhanced mixing are quite beneficial for the overall efficiency of radiolabelling reaction processes. Radiolabeling carried on microfluidic reactors usually results in purer products, higher yields, greater selectivities, and shorter reaction times than conventional methods. Furthermore, more benign and milder reaction conditions could be applied for certain reactions within microfluidic devices, which is very helpful for maintenance of the bioactivities of some peptides.

## 4. Preparation of Peptide-Based PET Tracers with Microfluidic Reactors

Conventional methods for radiolabeling of peptides have several limitations. (1) Large excess of precursors are needed to promote rapid and efficient labeling, and then extensive purification is required in order to separate the product from precursors; otherwise these cold precursors would occupy the targets and result in lower imaging quality. (2) Strict reaction conditions, which are hard to bear for some peptides, are needed, especially in the case of F-18 labeling. It would be promising to apply microfluidics technology to prepare peptide-based PET tracers. 

**Figure 4:**
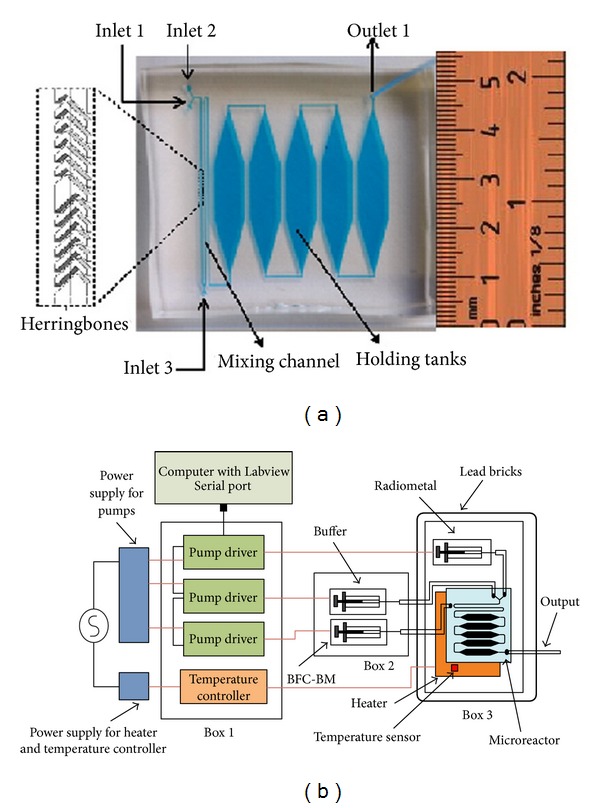
Optical micrograph of the microreactor (a) and schematic of the microreactor system for radiolabeling (b). (Reproduced from [4] with permission from Elsevier).

Recently, a PDMS microreactor was fabricated and tested for the labeling of bifunctional chelate conjugated biomolecules with PET radiometals ^64^Cu and ^68^Ga in aqueous solutions (Figure 4) [4, 41]. The results showed that the microfluidic approach overall outperforms conventional radiosynthetic methods. The PDMS microreactor had a serpentine microchannel for mixing, a series of reservoirs for the incubation of the radiometal-ligand mixture, and a thin-film heater for heating the mixture. The reservoir was composed of 5 hexagonal chambers connected in series, with a total volume of 50 *μ*L. DOTA-RGD conjugate was labeled with ^64^Cu at mild reaction condition (23–47°C, 5–20 minutes). The incorporation yield is considerably better (~90%) than that obtained in classical vessel radiochemistry (~60%). The authors further investigated radiolabeling of both DOTA-peptides and NOTA-peptides conjugate with ^68^Ga, and similar conclusions were drawn. These results demonstrated that it was possible to achieve high radiolabeling yields without using excess of peptide precursors, and this would eliminate the need for chromatographic purification of the product to remove unlabeled peptides. 

Synthesis and purification methods of the widely used prosthetic group ^18^F-SFB based on microreactor have been developed [42]. It was a good example for multistep synthesis and purification of radiopharmaceuticals for PET. Aqueous ^18^F-fluoride (100–500 MBq) was concentrated and further eluted to a microreactor for evaporation. ^18^F-fluorination of the precursor (1.5 mg) was carried out at high temperature (200°C for 4 minutes), followed by hydrolysis and subsequent activation of the 4-^18^F-fluorobenzoyl group. Purification was performed on a miniaturized solid phase extraction column. It took about 25 minutes, and ^18^F-SFB was obtained with 55 ± 6% yield (not decay-corrected) and >98% radiochemical purity. 

Radiolabeling of peptides with ^18^F-SFB utilizing microfluidic technology was reported [43]. In their study, ^18^F-SFB was firstly synthesized by conventional method and concentrated to a final volume of 50~100 *μ*L in CH_3_CN for further use. Then two different approaches were applied for the radiolabeling of the phosphopeptide-cell-penetrating peptide dimers: conventional labeling and microfluidic technology. The isolated radiochemical yields using microfluidic technology (~26%) were much higher than those using conventional labeling methods (2% to 4%). And it was also found that the *N*-terminal acylation of ^18^F-SFB was more selective in the microfluidic-based reaction compared to the conventional radiolabeling procedure. Liu et al. reported a labeling system for biomolecules via ^18^F-SFB utilizing a digital microfluidic droplet generation (DMDG) chip which allowed rapid scouting of reaction conditions in nanoliter volumes [44]. This system required only very small amounts of precursors (200- to 2000-fold reduction than conventional method). And it might be utilized for radio-labeling of a diverse spectrum of biomolecules including intact antibodies and their fragments, other proteins, and peptides.

Direct radiolabelling of peptides with ^18^F-fluoride was also successfully achieved using the Advion NanoTek continuous flow microreactor [45]. These results showed radiochemical yields were dependent on the leaving group, precursor concentration, reaction temperature, and flow rate. The optimal temperature in the microreactor was 70–80°C yielding labeling efficiency up to 90%. They had some very promising findings: (1) the reaction progressed even at 35°C with 30–40% labelling yields; (2) reactions could be performed even at a concentration of 0.5 mg mL^−1^ with reasonable yields. These results demonstrated that the microreactor may be used for labeling of thermally labile peptide molecules with ^18^F radioisotope under very mild conditions.

## 5. Conclusion

Peptide-based PET tracers are valuable tools for peptide receptor imaging in clinical oncology and a large variety of radiolabeled peptides analogues have been developed for *in vivo* detection of tumors overexpressing relevant receptors. And many of them were under clinical and preclinical investigation. Large-scale clinical studies are still relatively challenging due to the complex radiochemistry procedures for peptide-based PET tracers, such as multistep synthesis and purification. Convenient radiolabeling technology, which could simplify the synthesis and purification procedures, is needed. In the last decade, remarkable progress has been achieved in the field of microfluidics-based PET radiochemistry. With the help of microfluidics system, rapid and efficient preparation of peptide-based PET tracers might be achieved. By now, some microfluidic systems have been explored for peptide radiolabeling, which may provide great versatility for the production of imaging agents in a doses-on-demand way for clinic use. In conclusion, microfluidics is a very promising technology to meet the increased demand for peptide-based PET tracers. 

## Figures and Tables

**Table 1:** Receptors for the peptide-based PET tracers [11–14].

Receptors	Peptide	Cancer	Labeling
Somatostatin receptor	Somatostatin	Neuroendocrine tumors	^ 68^Ga-DOTA, ^68^Ga-NOTA, ^64^Cu-TETA, ^64^Cu-DOTA, ^64^Cu-NOTA, ^64^Cu-CB-TE2A, ^18^F-NFP, ^18^F-SFB, and so forth
Gastrin-releasing peptide receptor (GRPR)	Bombesin	Prostate cancer, breast cancer, Gastrointestinal stromal tumor	
*α*_*v*_ *β*_3_-integrin	RGD	Brain cancer, lung cancer, breast cancer, and so forth	

Melanocortin 1 receptor (MC1R)	*α*-MSH	Melanomas	^ 18^F-SFB, ^68^Ga-DOTA, ^64^Cu-TETA, ^64^Cu-DOTA, ^64^Cu-CB-TE2A

Cholecystokinin B/gastrin receptor (CCK_2_/CCK-B)	CCK/gastrin	Medullary thyroid cancer	^ 18^F-SFB, ^68^Ga-DOTA, ^64^Cu-DOTA

Glucagon-like peptide-1 receptor (GLP-1)	Exendin	Insulinoma cancer	^ 18^F-FBEM, ^68^Ga-DOTA

Neurotensin receptor (NTR1)	Neurotensin	Small cell lung cancer, colon cancer, and so forth	^ 68^Ga-DOTA ^18^F-FB

Neuropeptide Y receptor (Y1)	NPY	Breast cancer, prostate cancer	^ 64^Cu-DOTA

Luteinizing hormone-releasing hormone receptor (LHRH-R)	LHRH	Prostate cancer, breast cancer, and so forth	^ 68^Ga-DOTA

Neurokinin 1 receptor (NK-1)	Substance P	Glioblastoma	

Vasoactive intestinal peptide **receptor** (VIP-R)	Vasoactive intestinal peptide (VIP)	Prostate cancer	^ 64^Cu-DOTA ^18^F-FB

Pituitary adenylate cyclase-activating peptide (PACAP) **receptor**	Pituitary adenylate cyclase-activating peptide (PACAP)	Breast cancer	^ 64^Cu-DOTA

Chemokine receptor 4 (CXCR4)	CXCR4	Lymphatic system, lung cancer, and so forth	^ 18^F-SFB, ^68^Ga-DOTA, ^64^Cu-DOTA

**Table 2:** Commonly used radioisotopes for peptide-based PET tracer.

Radionuclide	Half-life	*E*_max⁡_ (*β* ^+^)/abundance	Production	Chemistry
^ 18^F	109.8 min	634 Kev/97%	Cyclotron	Organic chemistry Chelation chemistry
^ 64^Cu	12.8 h	656 Kev/19%	Cyclotron	Chelation chemistry
^ 68^Ga	67.6 min	1899 Kev/89%	Generator	Chelation chemistry
